# Patient perceptions of integrated care: confused by the term, clear on the concept

**DOI:** 10.5334/ijic.985

**Published:** 2013-03-08

**Authors:** Kara Odom Walker, Alanna Labat, Judy Choi, Julie Schmittdiel, Anita L. Stewart, Kevin Grumbach

**Affiliations:** Department of Family and Community Medicine, University of California, San Francisco, CA, USA; Department of Family and Community Medicine, University of California, San Francisco, CA, USA; Department of Family Medicine, University of California, Los Angeles, CA, USA; Kaiser Permanente Northern California Division of Research, Oakland, CA, USA; Institute for Health & Aging, Center for Aging in Diverse Communities, University of California, San Francisco, CA, USA; Department of Family and Community Medicine, University of California, San Francisco, CA, USA

**Keywords:** patient centered experiences, integrated care, framework

## Abstract

**Purpose:**

Health care reform in the US has introduced terms such as ‘the patient-centered medical home’ and ‘integrated care’ that are often unclear and unfamiliar to patients. This study explored patient experiences with the functional domains of integrated care.

**Theory and methods:**

Patients first wrote their definitions of integrated care and then participated in focus group discussions about their experiences with the health care system. Transcripts were analyzed for thematic content.

**Results:**

Forty-four patients participated in one of seven focus groups in San Francisco, CA in English and Spanish. Many patients were not clear about the meaning of the term integrated care. However, patients described experiences largely reflected in an existing conceptual model of integrated care and the importance of coordination within and across teams and with community resources, continuity and sharing of information, and patient engagement. Patients with high medical needs described the ubiquitous challenges they faced in experiencing coordinated care.

**Conclusions:**

Patients may not understand the term integrated care but are relatively clear on what the concept of integrated care entails and support its successful implementation. Patients and their families are at the center of integrated care, and health systems need to support and empower them to successfully navigate the medical neighborhood.

## Purpose

High quality primary care medical homes are necessary but not sufficient to achieve effective and efficient health care delivery. Patients also need well-functioning medical neighborhoods as well as medical homes [1]. The medical neighborhood is a term coined by Fisher to describe the constellation of services, providers and organizations in a health system that contributes to the care of a population [2]. In addition to primary care medical homes, the medical neighborhood consists of specialists, emergency facilities, inpatient services, home care, pharmacies, and other components. Coordination among the elements of the medical neighborhood, including the primary care medical home, is important for meeting patients’ comprehensive health care needs and promoting health equity [3–5].

Policies and incentives promoting formation of accountable care organizations in the US seek to achieve more integrated care (i.e. more coordinated) within medical neighborhoods built on a foundation of primary care. Other nations with public financing of universal health care coverage also face challenges in overcoming traditional silos in the health care sector, such as between hospital and ambulatory care or primary care and specialty care, and assuring that comprehensive financial coverage translates into well-coordinated delivery of care to individual patients. As groups of healthcare providers merge into accountable care organizations to provide more coordinated care and chronic disease management, the goal of providing improved quality and cost has moved the conversation of integrated care experiences forward. This growing interest in integrated care is raising many of the same types of questions about definitions of terms and concepts that have previously surfaced about primary care, the primary care medical home and now, the ‘patient-centered medical home’. This term refers to a care model that encompasses the traditional tenets of primary care, such as accessible, comprehensive, coordinated care, and augments this model with a greater emphasis on patient-centeredness, innovative practice approaches, and reformed payment methods that better support primary care [6]. Many patients may be unclear about the terms ‘primary care’ and ‘patient-centered medical home.’ Patients have described the patient-centered medical home in the continuum of ‘parent’s home, nursing home and funeral home’ [7]. Yet when encouraged to express in their own words what they desire in health care, patients articulate the importance of the core functional elements of primary care such as accessibility, continuity, and coordination [8]. These elements also need to be present in the patient-centered medical home and into the medical neighborhood.

Similarly, the term ‘integrated care’ is not transparent to patients or providers. In the US, integrated care is often used to mean structural integration of facilities and providers, as in vertically integrated delivery systems or horizontally integrated hospital chains. Integrated care is also used to refer to colocation of services, such as deploying mental health and primary care providers at a single site. However, what is most critical to a medical neighborhood is functional integration in patient care irrespective of ownership and location. Because of imprecise terminology, there are no commonly accepted metrics for ‘integrated-ness’ that could be used to advance research, practice and policy in this area, despite the prominence of this concept in policy and practice changes throughout health systems in North America, Europe and other parts of the globe. The lack of standard metrics reflects what has been described as the ‘elastic’ and ‘bewildering’ terminology and conceptual models of integrated care [9]. The patient voice should be part of the conversation about the meaning of integrated care [10].

We conducted a series of focus groups with an ethnically diverse sample of patients to explore their understanding and experiences of integrated care. Our first aim was to investigate what the term integrated care meant to patients. Our second aim was to explore their experiences with the functional domains of integrated care based on a definition recently proposed by Singer: “patient care that is coordinated across professionals, facilities, and support systems; continuous over time and between visits; tailored to the patients’ needs and preferences; and based on shared responsibility between patient and caregivers for optimizing health” [11] Singer’s framework has been developed from a synthesis of both US and international-based definitions and frameworks. It includes five domains related to coordination and two to patient-centeredness (Table 1).

## Theory and methods

We conducted seven focus groups with a total of 44 patients. To recruit patients likely to have relatively high care coordination needs, we used the following inclusion criteria: 50 years of age or older, one or more chronic conditions (diabetes, hypertension, chronic lung disease, depression, chronic kidney disease, osteoarthritis, congestive heart failure, or mild cognitive impairment), at least two medical visits in the past 12 months, and fluency in English or Spanish. Patients were recruited from a large integrated delivery system, county-administered primary care clinics, and primary care clinics at an Academic Health Center, all located in the San Francisco Bay Area. Focus group participants were grouped by practice setting and language (6 in English, 1 in Spanish). Focus groups were conducted between May 2011 and September 2011. The UCSF Institutional Review Board approved the study protocol.

### Focus group protocol

While waiting for the focus group to start, participants were asked to write down their answer to the question, ‘What is integrated care?’ (‘¿Que significa ‘atencion integral’ para usted?’ in Spanish). Interviewers trained in the use of qualitative interview techniques then facilitated a discussion using a series of open-ended questions.

The interview guide was developed based on an extensive literature review of integrated care concepts, most of which fell under the domains delineated by Singer. After they completed the written question about integrated care, we asked the group to verbalize their overall notion of ‘integrated care’. We then asked patients to describe their health care experiences in multiple settings. We also asked about their satisfaction, opinions about how doctors and other caregivers should ‘share information’ and ‘work together,’ and their views of coordinated and integrated care including care received in primary care, specialty, laboratory, inpatient and emergency room settings. We also asked about other relevant health care experiences not specifically addressed by our questions or contained in the Singer conceptual model.

### Data analysis

We first compiled all of the written definitions into a list and categorized them into related themes and subthemes. Recorded interviews were transcribed and the transcripts reviewed by the interviewer for accuracy. Two research team members (K.W. and A.L.) independently analyzed each transcript using qualitative content-analysis methods to identify meaningful quotes. Atlas.ti software version 5.2 (Atlas.ti Scientific Software Development, Berlin, Germany) was used for data management and analysis.

Transcripts were read several times in an iterative process to identify recurring concepts that represented distinct domains of integrated care. These concepts were signified by codes used to label quotations that represented discrete thoughts. We continued to add codes that represented meaningful ideas until all transcripts were coded in their entirety. After independently coding transcripts, between-coder comparisons were completed and the codes were compiled into a revised codebook that included the comprehensive list of codes. Our Kappa calculation of 83% indicated high levels of inter-rater agreement. Coded quotes were assembled into larger categories or themes. Within each theme, we then explored subthemes that emerged within each larger theme. Within each subtheme, we selected representative statements based on relevance and clarity of expression. Three investigators (K.W., A.L., and K.G.) then reviewed the sub-themes for relevancy and consistency. After independently creating our own subthemes and themes, we then compared our findings to existing frameworks of integrated care.

## Results

The 44 focus group participants were mostly between the age of 50–64 years old (64%) and diverse in their ethnicity and level of education (Table 2). Most had a usual source of care (94%) and had seen a specialist in the past two years (73%).

### Understanding of the term integrated care in written responses

Analysis of written definitions indicated that patients varied substantially in terms of how they defined the term ‘integrated care’. The number of definitions provided by each patient ranged from 0 (did not respond) to 4, for a total of 97 definitions. Some patients provided at least one definition that was consistent with that proposed by Singer. For example, 67 definitions pertained to collaboration, information sharing, coordinated care, and a medical home, thus capturing the essence of integrated care. In the words of one patient, ‘To me it means that all parties involved are working as a whole as everything that goes on is shared with everyone.’ Another patient wrote that integrated care is ‘a system that has components working together.’

Out of all the offered definitions, a majority were related to Singer’s domains or subdomains, although the definitions did not include the entirety of dimensions for the term. However, another 30% of patients did not provide a definition that fit within our working framework.

Many patients defined the term in a way that indicated a lack of knowledge about the concept. Some thought integrated care was related to integrative medicine (e.g. ‘integrate mind-body-soul’), self-care, or with racial integration (‘everyone should be treated equally’). Several patients in the Spanish-language group interpreted the concept as meaning eating whole foods; ‘atencion integral,’ the term we used in Spanish, is frequently used to describe whole grains. This misunderstanding shows that the term integrated care cannot be used with patients to reliably convey overarching goals of integration. With some additional prompts, those patients may have been able to further describe aspects of integrated care that were related, but a priori did not have any preconceived related definitions.

### Understanding the concepts of integrated care: focus group discussions

Analyses of the focus group transcripts resulted in themes and subthemes that organized along the same domains as described by Singer et al. The details pertaining to each of the seven categories from our initial conceptual framework correspond to Table 1. Table 3 summarizes the subdomains and provides example quotes, organized by the categories.

#### Theme 1: Coordination within care team

Many patients described how coordination must occur within the care team. Patients wanted providers to know their medical history and care plan, regardless of the medical provider who knows them best. Patients described their frustration in having to repeat information and in receiving conflicting information. They also noted that duplication of effort sometimes occurred as a result.

#### Theme 2: Coordination across teams

Patients noticed when the information about their medical history, referrals, or treatment plans was not shared with other providers and care sites. Many patients did not understand why they were often expected to be the expert for their own medical conditions, despite not fully understanding medical jargon and details about diagnoses and past treatments. Other patients noted that physicians who were providing care in other settings should just contact their personal doctor, as they were aware that no attempt was made to contact their personal doctor.

#### Theme 3: Coordinated between care teams and community resources

Several patients expressed appreciation when their primary care teams and other providers helped to facilitate connections to community based resources. These linkages often provided additional resources and support that patients otherwise would not have had. Community based resources could provide additional support, medical supplies, and even transportation.

#### Theme 4: Continuous familiarity with patient over time

The longitudinal knowledge of the patient’s medical history across healthcare settings also emerged as an important theme. Continuity has long been an important part of the primary care experience [11]. Patients expressed the need for their doctors and other providers to know their history. Patients described the sense of continuous familiarity as involving both a longitudinal relationship with a primary care physician and information sharing across settings and time. Some patients carried their records on USB or in a portable folder; others had a web-based health portal. Other patients desired consistent communication between providers and the patient regardless of whether this occurs during an office visit, on a phone call, or email. Relying on the medical charts alone is often fraught with error and incomplete or missing information. Patients shared multiple examples of how they observed that the charts had missing parts of their medical history, current medication lists or care plan.

#### Theme 5: Continuous and proactive and responsive action between visits

Patients expressed a desire for responsiveness to their concerns and questions, regardless of whether they contacted the primary care clinician, specialist, lab, or billing office. Patients wanted to be able to get appointments, set up follow-up appointments and address insurance questions without significant barriers or delays. Patients described how they need access not only at the time of appointments, but also between doctors’ visits. The manner in which their needs were addressed could occur through email, phone, internet health portals, or written communication. Using these modes of communication in an effective manner allowed patients to feel that their doctor was checking on their ongoing healthcare needs. Those who had access to medical record web-portals to review test results and communicate with their office staff or doctor were generally very pleased with this tool. Some patients described difficulty communicating with their providers outside of visits and tried to find ways to facilitate ongoing conversations for their healthcare questions, needs and updates.

#### Theme 6: Patient-centered care

Patients expressed satisfaction with care that was responsive to patient preferences in a respectful, culturally sensitive and supportive environment. They also described their frustration when care was not centered on their needs or experiences. Patients also described feeling that doctors and hospitals were focused on provided care to them as they needed it so that they could be empowered to take care of their own health care needs. With information availability, continuous conversation, and ongoing decision making around their individual needs, patients felt that the healthcare system was working on their behalf, rather than for other reasons (i.e. profits, convenience).

#### Theme 7: Shared responsibility

Patients described integrated care as health teams and patients working together to improve their health and manage available resources. Patients wanted to be empowered, along with their family and at-home caregivers, in medical decision-making and care coordination. Many patients acknowledged their role as part of the health care team, whether it was having labs ordered, test results returned, or appointments scheduled. At the same time, some patients communicated a sense of burden about the degree of responsibility they shouldered for coordinating various aspects of their care. They described creative strategies to navigate the system through helpful office staff, savvy family members, or personal connections. Some described experiences of care so disjointed that they had to take responsibility for their own longitudinal record of care and manage all parts of their health care needs. Even when a patient has a responsive, patient-centered medical home, patients pointed out that it is the patient who lives 24/7 with his or her medical conditions and often must deal with a myriad of care coordination needs.

## Discussion

Our study underscores the importance of appreciating patients’ perceptions of the meaning of integrated care. Similar to investigations of patients’ understanding of patient-centered medical homes [7, 8, 12], our exploration of the term ‘integrated care’ indicates that patients do not understand the term but are relatively clear on the concept. Out of all the offered definitions, a majority were related to our domains or subdomains. The definitions did not include the entirety of dimensions for the term. Another 30% of patient did not provide a related definition to our working framework. With some additional prompts, those patients may have been able to further describe aspects of integrated care that were related, but a priori did not have any preconceived related definitions.

Our results suggest that the jargon-laden terms of integration and coordination may not be patient friendly. While these terms may serve ably as code words in communication among experts in the field, they are unlikely to function well in efforts to communicate with patients and the public about delivery system reform and the goals of accountable care organizations and other related reforms.

Our study indicates that patients often can perceive when integration and coordination are—or are not—happening in their experiences with the health care system. In their own words, patients used open-ended descriptions that focus on information sharing across personnel, sites, and time, along with shared responsibility and a sense of all members of the care team ‘being on the same page.’ Although their descriptions during the discussions sometimes included positive experiences when the system worked well, most patients found it easier to describe how poor integration and coordination resulted in negative experiences. These negative experiences stood out in their memory and were described as duplicated effort and conflicting information about their medical history and care plan. The results of poor coordination often made them feel they wasted their time or did not follow a care plan as directed.

Our findings strongly support the conceptual framework of integrated care developed by Singer. The themes that emerged from the focus groups largely aligned with the definitions of the seven domains of this conceptual model and no major new domains were identified.

Our results allow us to provide a more detailed definition of each of the domains than provided by Singer. For example, in discussing access to care, patients in the focus groups tended to talk not only about access between visits but also about access to appointments and visits. The domain ‘proactive and responsive action between visits’ needs to be expanded to include responsive actions in visit scheduling and attendance. Our findings can inform more systematic efforts to measure and evaluate integrated care that captures the patient experience. An important next step is testing and validating survey instruments to quantitatively measure these domains of integrated care; these instruments could then be used to assess the impact of delivery system reforms on patients’ experiences of integrated care.

Our study also indicates that the domains of integrated care and primary care are complementary. Most of the domains that patients described as important for integrated care, including continuity, coordination, access, and comprehensive services, are also core domains of primary care [13–17]. In essence, our study suggests that integrated care can be conceptualized as a health systems property and primary care as a key component of that system. While primary care plays a unique and influential role in integrating care, and for most patients is a necessary element for integrated care, patients clearly desire all components of the health care system to be patient-centered and work together. Patients articulated a sense of the interdependency of the various components of the medical neighborhood.

We were also struck by the extent of the care coordination burden shouldered by patients in the focus groups with extensive medical needs. This burden included not just the types of major challenges in coordination that are now receiving attention from formal care management programs, such as coordination in the transition from a hospital stay to home. Patients described the more mundane but ubiquitous difficulties they often experienced in refilling prescriptions, arranging visits to specialists and allied health services, obtaining medical equipment, and transmitting information. Patients and their families are at the center of integrated care, and medical homes and health systems need to support and empower them to successfully navigate the medical neighborhood. For the patient experience of integrated care to improve, we must consider all of their interactions with the healthcare system, particularly for those who have multiple chronic conditions. These patients would benefit most from information coordination and communication across sites of care. Meaningful use of health information technology may improve information sharing and coordination, but our findings suggest that it will only be part of the solution to enhancing patients’ experience of integrated care.

There are several limitations to our study. Though useful for exploring themes, focus groups can be susceptible to respondent and researcher bias and subjective interpretation. We attempted to reduce potential bias through independent reviews of the data by different members of the study team. Patients included in the focus groups were not chosen at random and their opinions may not be representative of those of the overall target population. A number of the participants were members of an integrated care system and may have more positive experiences with integrated care than patients cared for in other settings. However, our themes quickly reached saturation with participants recruited across different practice settings. We targeted patients with ongoing health care needs, and the findings may be less applicable to populations without chronic medical conditions who mainly seek episodic care.

In conclusion, our study emphasizes the need for continued work in patient-centered communication strategies for evaluation and monitoring of health delivery system reform. Although many patients do not appreciate the full meaning of the term integrated care, most are relatively clear on the concept and value coordinated care across the medical neighborhood. We could have explored with the participants a substitute term for integrated care that would represent a ‘catch-all substitute’ for integrated care. A term other than coordination is needed to communicate the concepts of integrated care among patient populations. A subsequent study could explore a patient-centered term for integrated care. Patients and their families are at the center of integrated care, and medical homes and health systems need to support and empower them to successfully navigate the medical neighborhood. Future research should validate instruments to more systematically measure patient experience of integrated care. This study can inform ongoing work that leverages the patient voice in future measurement development.

## Acknowledgments

We would like to acknowledge patients who participated in our focus groups, and our advisory board members, including Thomas Bodenheimer, MD, MPH, Paul Grundy, MD, MPH, Wendy Jameson, MPH, MPP, Eliseo J. Perez-Stable, MD, Phil Madvig, MD, Diane Rittenhouse, MD, and Hal Yee, MD, PhD.

## Financial Support

This study was supported by a grant from The Aetna Foundation (grant number 4051077), a national foundation based in Hartford, Connecticut that supports projects to promote wellness, health and access to high quality health care for everyone. Additional support was provided by a grant from the National Institute on Aging (grant P30AG015272) to the UCSF Center for Aging in Diversity Communities and by the National Center for Research Resources and the National Center for Advancing Translational Sciences, National Institutes of Health, through UCSF-CTSI Grant Number UL1 RR024131. Dr. Schmittdiel was supported by the Health Delivery Systems Center for Diabetes Translational Research (CDTR) [NIDDK grant 1P30-DK092924]. The contents are solely the responsibility of the authors and not necessarily those of The Aetna Foundation, its directors, officers, or staff and do not necessarily represent the official views of the NIH.

## Conflict of interest statement

The authors have no conflicts of interest to declare.

## Reviewers

**Matthew Harris**, DPhil, MBBS, MSc, MFPH, Clinical Lecturer in Public Health, Department of Primary Care and Public Health, Imperial College London, UK W6 8RP

**Rebecca Malouin**, Phd, MPH, Assistant professor in the Department of Family Medicine and Department of Pediatrics and Human Development at the Michigan State University College of Human Medicine, USA

**Laurann Yen**, Research Fellow, Australian Primary Health Care Research Institute, Australian National University, Ian Potter house, Cnr Marcus Clarke and Gordon streets, Acton 0200 Canberra, Australia

## Figures and Tables

**Table 1.**
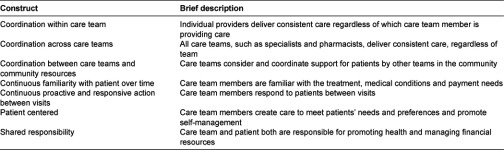
Conceptual framework of integrated patient care based on Singer et al. [11].

**Table 2.**
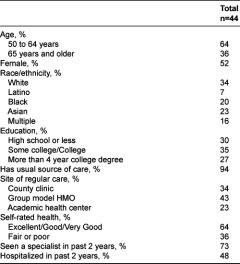
Focus group participant characteristics.

**Table 3.**
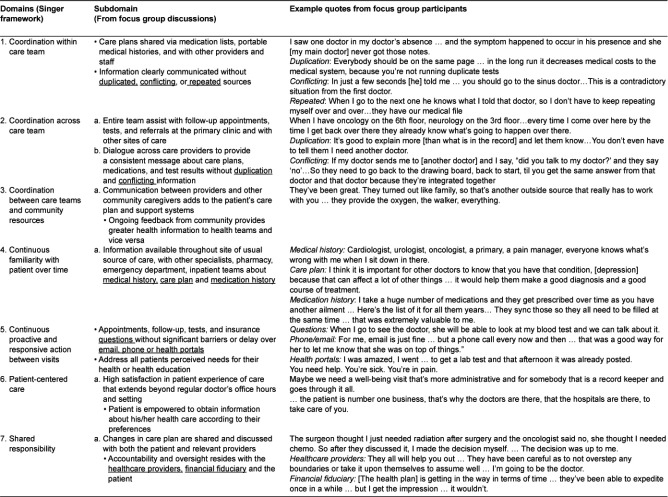
Domains and subdomains with sample quotes from focus group discussions.
